# Boron Carbide as an Electrode Material: Tailoring Particle Morphology to Control Capacitive Behaviour

**DOI:** 10.3390/ma16020861

**Published:** 2023-01-16

**Authors:** Suna Avcıoğlu, Merve Buldu-Akturk, Emre Erdem, Figen Kaya, Cengiz Kaya

**Affiliations:** 1 Department of Metallurgical and Materials Engineering, Faculty of Chemistry and Metallurgy, Davutpaşa Campus, Yildiz Technical University, Istanbul 34210, Turkey; 2 Faculty of Engineering and Natural Sciences, Materials Science and Nano Engineering, Sabanci University, Istanbul 34956, Turkey; 3 Sabanci University Integrated Manufacturing Technologies Research and Application Center, Composite Technologies Center of Excellence, Teknopark Istanbul, Pendik, Istanbul 34906, Turkey

**Keywords:** boron carbide (B_4_C), morphology, supercapacitor, electrode, energy storage

## Abstract

In this study, boron carbide powders consisting mainly of nano/micro fibers or polyhedral-equiaxed particles were synthesized via the sol–gel technique, and the influence of particle morphology on electrochemical performance of boron carbide electrodes was investigated. Thermal decomposition duration of the precursors played a determinant role in the final morphology of the synthesized boron carbide powders. The morphology of boron carbide powders successfully tuned from polyhedral-equiaxed (with ~3 µm average particle size) to nano/micro fibers by adjusting the thermal decomposition duration of precursors. The length and thickness of fibers were in the range of 30 to 200 µm and sub-micron to 5 µm, respectively. The electrochemical performance analysis of boron carbide powders has shown that the particle morphology has a considerable impact on the boron carbide electrodes electrochemical performance. It was found that the synergetic effects of polyhedral-equiaxed and nano/micro fiber morphologies exhibited the best electrochemical performance in supercapacitor devices, resulting in the power and energy density of 34.9 W/kg and 0.016 Wh/kg, respectively.

## 1. Introduction

The necessity of increasing the use of renewable energy sources instead of fossil fuels is no longer a matter of debate. To reduce fossil fuel consumption, the efficient production of environmentally friendly energy, as well as developing safe storage systems for harvested energy, is an urgent need. Supercapacitors (SCs) are among the most promising energy storage devices, exhibiting fast charge–discharge characteristics. Therefore, they are often employed in applications where quick energy storage and release is required, such as start–stop power supply in hybrid automobiles and wind turbines, due to their fast charge–discharge characteristics, extended cycle life (>10^5^ cycles), and high-power density [1,2]. Moreover, the safety issues of batteries and extended cycle lives (>10^5^ cycles) of SCs makes the latter more reliable for wearable and portable electronics [3,4,5,6,7,8]. Nevertheless, the relatively lower energy density of SCs prevents their widespread application [9].

In SCs, the electrode material is key in defining the charge storage mechanism. Therefore, ample studies focused on developing novel electrode materials to improve the energy density of SCs. (I) Electric double-layer capacitors (EDLCs), (II) pseudocapacitors, and (III) hybrid capacitors are three different types of SCs, exhibiting different charge storage mechanisms [10,11,12]. The former relies on the interaction of electrolytic ions with electrode surface by electrostatic forces, resulting in the storage of electric charge at the electrode/electrolyte interface. Thus, good electrically conductive materials with a high surface area are desired to achieve high capacitance values of EDLCs. In this respect, carbon-based materials with varying morphologies, such as nanosheets, nanotubes, and fibers, have been comprehensively investigated for EDLCs [13,14,15,16,17]. On the other hand, capacitance generation in pseudocapacitors are based on reversible Faradaic processes [18]. In contrast to the batteries, Faradaic charge transfer processes among the electrolyte ions and electrode materials occur at the surface/near-surface of electrode material of pseudocapacitors. Thereby, charge transfer kinetics in pseudocapacitors is not restricted by solid-state ion diffusion, providing pseudocapacitors a relatively faster charge–discharge rate than batteries. Nevertheless, they are typically limited by their low energy densities [19]. Conducting polymers and transition metal oxides, such as MnO_2_, NiO, Co_3_O_4_, and ZnO, are commonly used electrode materials in pseudocapacitors [3,14,20,21]. In addition to these materials, recent studies show that polyoxometalate materials are also promising electrode materials for pseudocapacitors [8,22,23]. Hybrid supercapacitors have an asymmetric electrode configuration that combines the Faradaic and non-Faradaic electrodes to achieve elevated capacitance and high energy density [19]. However, the difference in charge transport rate of different types of electrodes needs to be overcome to reach further advancement in hybrid supercapacitors.

In recent years, carbide and nitride ceramics as electrodes for supercapacitors have also drawn intensive attention. For example, silicon carbide (SiC) nanowires fabricated by the low-pressure chemical vapor deposition (LPCVD) technique exhibited great potential as electrodes for micro-supercapacitors. It was found that silicon carbide nanowires demonstrate relatively higher capacitance value (~240 µF cm^−2^) than their thin-film counterparts [24]. The capacitance of silicon carbide nanowires was also further increased to 845 mF cm^−2^ by coating the nanowires with Co_3_O_4_ nanoflower clusters [25]. Boron nitride (BN) is another wide bandgap semiconductor material that has been used to fabricate battery and supercapacitor electrodes. M. Minakshi et al. developed BN-added MnO_2_ cathode material for alkaline batteries [26]. An electrode fabricated by combining hexagonal BN nanosheets (h-BNNSs) and reduced graphene oxide (rGO) provided robust and stable cycling performance [27]. C. K. Maity et al. introduced a BN nano-framework containing Zn-doped cadmium sulfide (Zn–CdS) and carbon nanotubes (CNTs) for asymmetric supercapacitors [28]. Recently, boron carbide (B_4_C) was also used in various electrochemical energy storage devices, such as lithium–oxygen, lithium–sulfur, vanadium redox flow, and zinc–air batteries [29,30,31,32]. Moreover, Chang et al. demonstrated that core–shell structure B_4_C@C can also be employed as an electrode material for all-solid-state micro-supercapacitors [33]. In addition, the roles of intrinsic defects in the electrochemical performance of B_4_C electrodes have also been studied [34]. Moreover, boron carbide has excellent chemical stability and low-volume expansion [35,36]. Even though it has relatively slower ionic transport kinetics, boron carbide has been indicated in previous studies to have great potential for use in energy storage devices [29,30,31,32,33]. Nevertheless, the influence of B_4_C morphology on its electrochemical performance has not yet been reported.

In this work, boron carbide powders with nano/micro fiber or polyhedral-equiaxed morphologies were synthesized via a low-temperature sol–gel synthesis route. Detailed structural analysis was also performed. The morphological properties were correlated with the electrochemical performance of the supercapacitor devices based on synthesized boron carbide. The supercapacitors were designed in symmetric and asymmetric ways, and the synergetic effects profoundly affected the capacitive properties positively. To our knowledge, the present study tests the electrochemical performance of boron carbide electrodes consisting mainly of nano/micro fibers, which were investigated and compared with fully polyhedral-equiaxed counterparts for the first time.

## 2. Materials and Methods

To obtain boron carbide particles with tailored morphologies, a non-catalytic sol–gel route that was previously reported by our group was modified [37,38,39]. Analytical grade glycerin (C_3_H_8_O_3_), tartaric acid (C_4_H_6_O_6_), and boric acid (H_3_BO_3_) were obtained from Merck and used without further purification. Synthesis procedure is as follows. Firstly, tartaric acid (7.5 gr) was added straight to glycerin (7.3 mL), and the mixture was stirred at 100 °C for 15 min using a magnetic stirrer until tartaric acid completely dissolved. Then, the solution added with boric acid (6.1 gr) and kept stirring for another 15 min at 120 °C. Afterwards, the reaction temperature raised to 150 °C during stirring, and the solution was kept at that temperature approximately 15 min until it transformed into a condensed gel. Following the gel synthesis stage, the condensed gel was cooled to ambient temperature, then thermally decomposed at 675 °C using a muffle furnace to remove excess carbon. Two samples were prepared by using 2 and 4 h thermal decomposition holding times and coded as 1Y and 2Y, respectively. Boron carbide particles with different morphologies obtained after the acquired 1Y and 2Y precursors heated at 1500 °C for 5 h in a tubular furnace under argon (500 mL/min). Fourier Transform Infrared Spectroscopy analysis (FT-IR, Bruker Tensor 27) was applied in the wavenumber range of 650–4000 cm^−1^ to characterize the chemical structures of 1Y and 2Y precursors. To determine the phase evaluation during heat treatments, thermogravimetric analyses (TG/DTG, Netzsch STA 44C) were carried out under argon from room temperature to 1450 °C with a heating rate of 5 ℃ min^−1^. Phase compositions of as-synthesized powders were analysed by X-ray diffraction technique (XRD, Bruker D2 Phaser), using Cu Kα radiation (λ = 1.540 Å, 30 kV, and 10 mA) in the range of 5° to 90° with a scanning speed of 1°/min. The microstructure of synthesized particles was inspected by a scanning electron microscopy (SEM, Zeiss EVO LS 10). The average dimensions of boron carbide particles were determined from SEM micrographs using Image-j software (version 1.5). Elemental point analysis was performed using an energy dispersive spectroscopy (EDS, Jeol-JED) detector attached to the SEM device.

All the electrochemical performance tests were performed in an aqueous solution of 6 M KOH by using a BioLogic VMP 300 electrochemical workstation with an impedance analyzer. Whatman glass microfibers were employed as the dielectric separator. As-prepared boron carbide powders with different morphologies were assembled into two-electrode supercapacitor devices. The devices consisted of two boron carbide electrodes on current collectors separated by an electrolyte-soaked separator. Electrochemical properties were analyzed using cyclic voltammetry (CV), potentiostatic electrochemical impedance spectroscopy (PEIS), and galvanostatic cycling with potential limitation (GCPL). CV curves were obtained at scan rates of 10 to 200 mV.s^−1^ from 0 to 1 V. PEIS analyses were performed in the frequency range of 10 MHz to 1 MHz, with an AC perturbation of 10 mV. GCPL curves were recorded at a scan rate of 10 mV/s within voltage window of −1 to +1 V at current densities of 0.10, 0.15, 0.20, 0.30, 0.50, and 2.40 A.g^−1^.

## 3. Results and Discussion

### 3.1. Sol–Gel Synthesis of Boron Carbide

**Figure 1:**
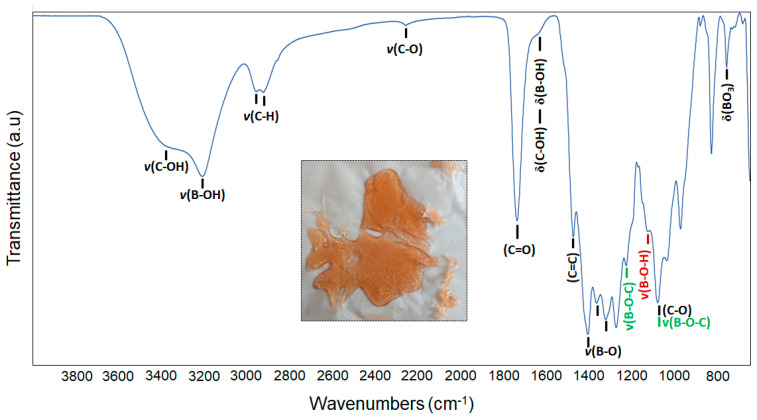
FT-IR spectra of the polyol-based condensed gel and its photograph (inset) taken at room temperature.

An amber-colored condensed gel was formed at the end of the first stage of the production process (Figure 1-inset). Figure 1 demonstrates the infrared spectra of the gel. The gel exhibits characteristic absorbance peaks of polyol-based condensed products at wave number regions consistent with previously reported studies [37,39]. Absorption bands associated with the C–OH and B–OH stretches are evident between 3000 and 3700 cm^−1^. The high intensity of these bands also indicates that the gel contains moisture. Well-resolved absorption bands belonging to C–H (2860–3000 cm^−1^) and C=O (1739 cm^−1^) stretching of carboxylic acid dimers are also apparent [40,41,42]. The bending modes of C–OH and B–OH are visible at approximately 1635 cm^−1^. The distinctive bands of B-O bonds can also be seen in the range of 1300–1500 cm^−1^. Absorption bands in the range of 760–650 cm^−1^ correspond to the bending vibrations of BO_3_ and B-O-O [43]. The peaks of B-O-C bonds (1231 and 1081 cm^−1^) confirm the formation of borate esters in the gel network through the dehydration and condensation reactions among starting materials [44,45,46].

**Figure 2:**
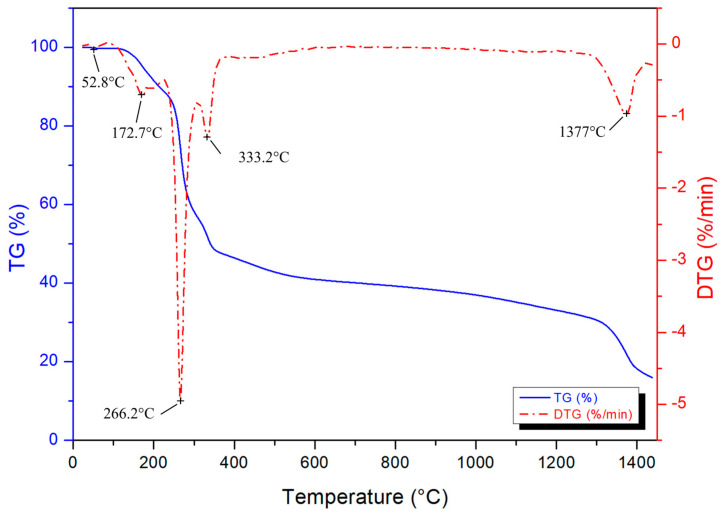
TG and DTG curves of the condensed gel.

Thermogravimetric analysis was performed for determining the thermal decomposition behaviour of the condensed gel and boron carbide formation temperature. The TG/DTG result is presented in Figure 2. The observed weight loss starts with the evaporation of physical water, followed by boric acid dehydration boron oxide (B_2_O_3_) from 80 °C up to approximately 230 °C [38,47,48,49,50]. The decomposition and combustion of the polymeric network occurs from 250 to 400 °C, resulting in a 48% weight loss. The following 12% weight lost might be ascribed to the reduction of carbon char. The last DTG peak is associated with the boron carbide phase formation, which starts at approximately 1250 °C, reaching to the maximum rate of seed formation at 1377 °C, and continuing up to 1450 °C.

**Figure 3:**
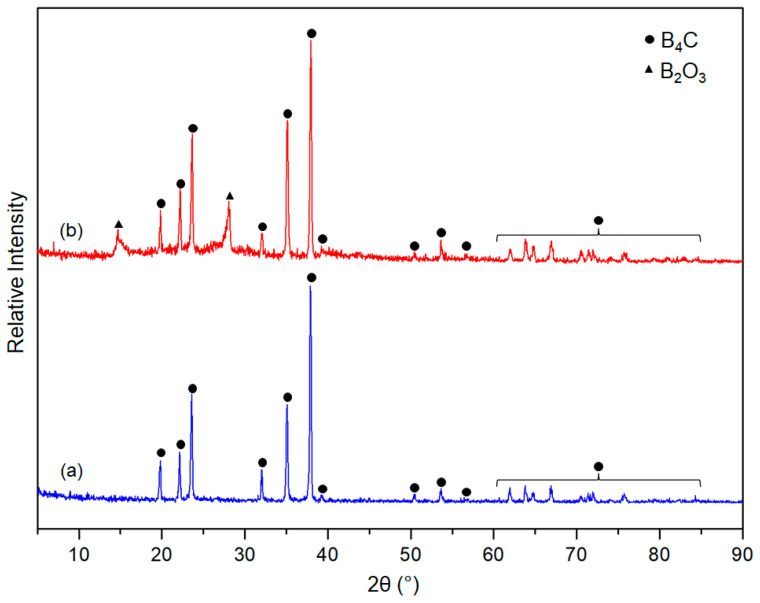
XRD patterns of as-synthesised powders obtained from (**a**) 2 h thermally degraded precursor and (**b**) 4 h thermally decomposed precursor.

X-ray diffraction patterns (XRD) of the synthesised boron carbide powders are shown in Figure 3. It is observed that the formed main phase in both powders is B_4_C (CoD: 96-412-4698) [38,39]. The results are also evident that boron carbide with high crystallinity could be obtained from both the two- and four-hour thermally degraded precursors. Nonetheless, the sample synthesized from the two-hour thermally degraded precursor was pure phase, but the sample derived from the four-hour thermally degraded precursor contains cubic B_2_O_3_ (CoD: 96-201-6173) as residual phase [51]. This phenomenon likely originated from the reduction of the carbon content in the four-hour thermally degraded precursor due to it relatively larger thermal decomposition duration.

**Figure 4:**
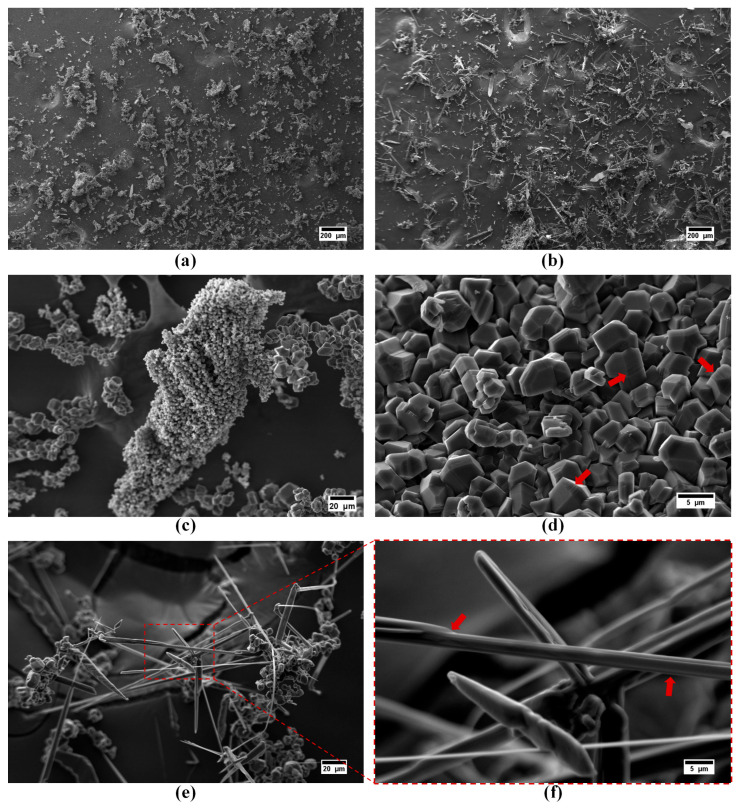
SEM images of the boron carbide as-synthesized powders derived from (**a**,**c**,**d**) 1Y and (**b**,**e**,**f**) 2Y precursors. The surface features of particles are emphasized with arrows.

The particle morphology of the synthesized boron carbide samples examined using scanning electron microscopy and the obtained micrographs are presented in Figure 4. Low magnification (100×) images of the powders were taken to present an overall morphological comparison. It is clear that adjusting thermal degradation duration of precursors significantly influenced the resulting morphology of boron carbides. Polyhedral-equiaxed morphology derived from the 2 h thermally decomposed precursor (Figure 4a). Contrarily, extending the thermal decomposition of precursor to 4 h resulted in the formation of boron carbide fibers (Figure 4b).

Higher magnification images of 1Y are presented in Figure 4c,d. It was found that the particles size distribution of polyhedral-equiaxed particles spanned a wide range. The majority of the polyhedral-equiaxed particles’ size is lower than ~5 µm, but few amounts of coarsened (~10 µm) polyhedral-equiaxed particles were also detected. The step formation during crystal growth on the polyhedral-equiaxed particles’ faces was observed and is emphasized with arrows in Figure 4d.

Higher magnification images of the sample obtained from 2Y precursor reveals that fibers have a monolithic structure, as shown in Figure 4e,f. Their lengths varied from 30 to 200 µm, whereas their thicknesses were in the range of sub-micron to 5 µm. The surface features of fibers (emphasized with arrows in Figure 4f) indicate that boron carbide fibers were grown with the lateral growth mechanism by the aid of liquid boron oxide droplets. Readers can refer to our previously reported study for more detailed information regarding the possible growth mechanisms of boron carbide fibers without the aid of any catalytic elements [39].

**Figure 5:**
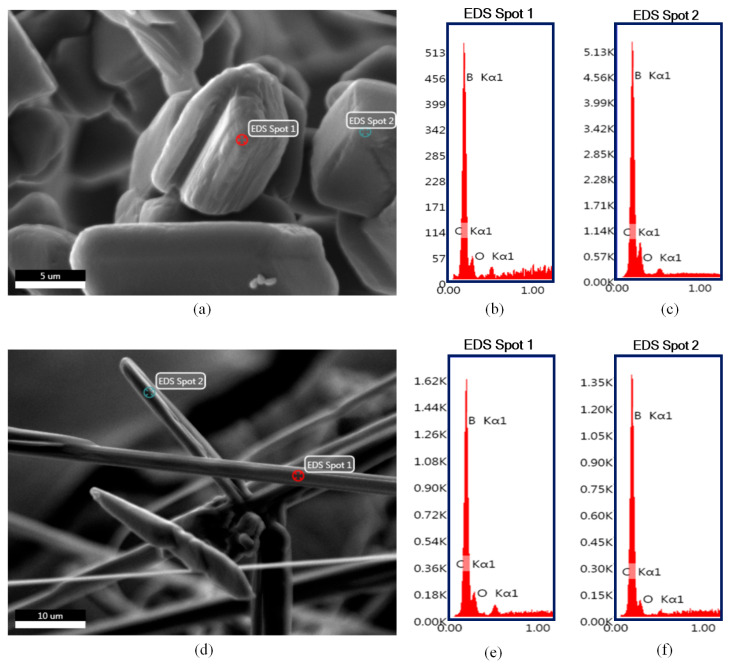
SEM-EDS point analysis results of the boron carbide as-synthesized powders derived from (**a**–**c**) 1Y and (**d**–**f**) 2Y precursors.

It is notable to mention that boron carbide powders synthesized from polymeric precursors via the sol–gel technique may contain residual graphite or amorphous carbon. In addition to XRD analysis results, these residual phases can easily be detected in SEM images due to their spongy-like morphology [38]. SEM images in Figure 4 and Figure 5 clearly indicate that there are no aggregates of residual graphite or amorphous carbon in the samples.

EDS point analysis results of the boron carbide particles with different morphologies are presented in Figure 5. It was found that regardless of their different morphologies, both polyhedral-equiaxed (Figure 5b,c) and fiber (Figure 5e,f) particles are boron carbide. The results also confirms that neither sample contains any elements other than boron, carbon, and oxygen. It is worth stressing that the presence of the oxygen signal might be attributed to the residual boron oxide phase as well as possible oxidation or hydration of boron carbide surfaces.

Our previous studies on the sol–gel synthesis of boron carbide showed that heat treatment temperature dramatically influences the final morphology, phase ratio, and particle size of synthesized powders. Heat treatment temperatures that are lower than 1500 °C were not sufficient to convert all the precursors to the boron carbide phase. It was determined that a high percentage of residual carbon-based phases remained in the as-synthesized powder produced under these conditions. In addition, due to the slow nucleation rate of boron carbide at low-heat treatment temperature, grain coarsening with a particle size exceeding 10 microns occurred. Due to these reasons, 1500 °C is chosen as the final heat treatment temperature. Further details on the effects of processing conditions on the characteristics of sol–gel-synthesized boron carbide particles can be found in our previously published studies [52].

### 3.2. Electrochemical Performance of Boron Carbide Particles

**Figure 6:**
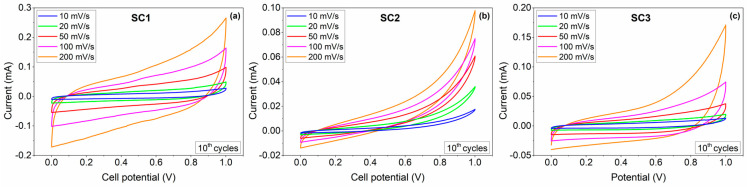
CV profiles of the symmetric (**a**) SC1 and (**b**) SC2 and the asymmetric (**c**) SC3 all-in-one supercapacitors at various scan rates in 6 M KOH.

**Figure 7:**
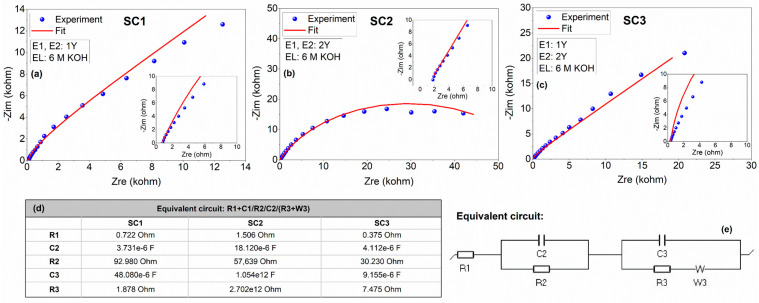
Experimental and fitted impedance data for the symmetric and asymmetric all-in-one supercapacitors in 6 M KOH electrolyte: Nyquist profiles of (**a**) SC1, (**b**) SC2, and (**c**) SC3; (**d**) fitted equivalent circuit elements of symmetric and asymmetric SCs; and (**e**) the equivalent circuit. The insets show high frequency regions of the spectra.

**Figure 8:**
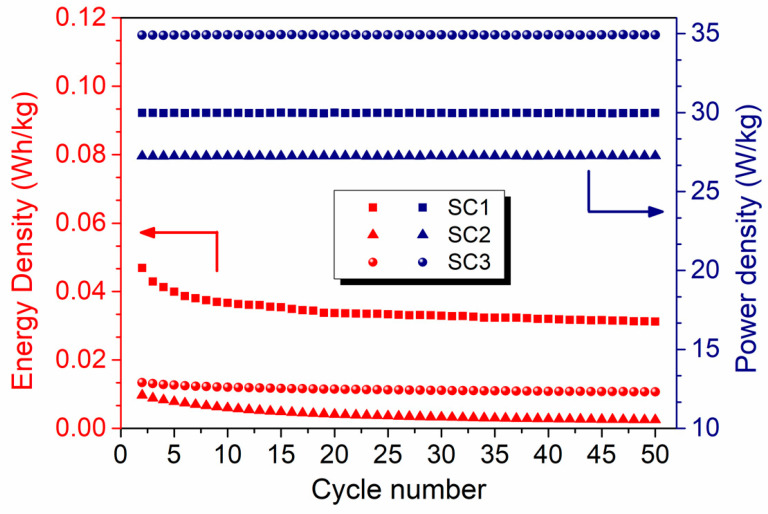
Comparison of the energy and power densities of the symmetric and asymmetric all-in-one SCs at a current density of 0.15 A.g^−1^.

**Figure 9:**
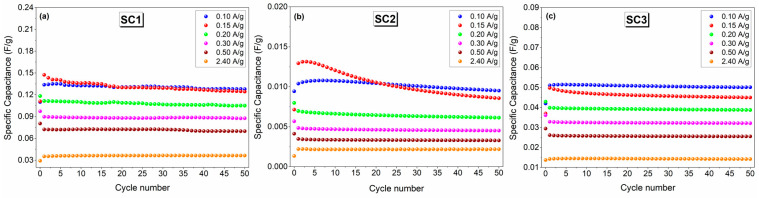
Comparison of the cycling performance of the symmetric and asymmetric all-in-one SCs at a current density of 0.15 A.g^−1^: (**a**) SC1, (**b**) SC2, and (**c**) SC3.

**Figure 10:**
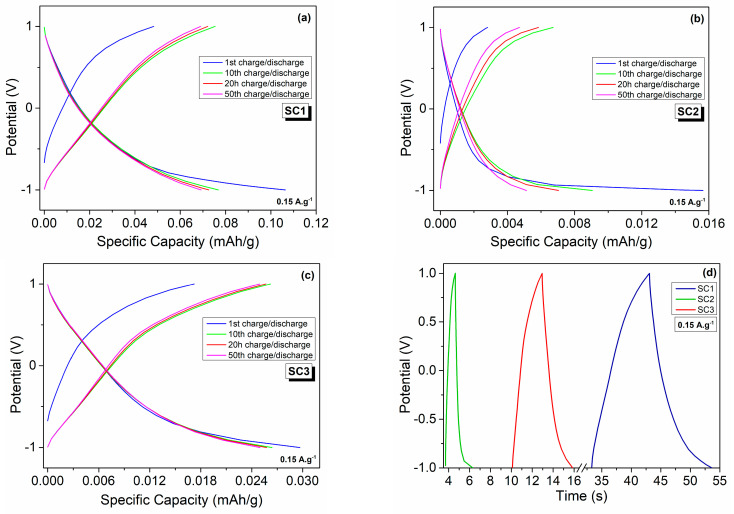
Galvanostatic charge–discharge profiles of the symmetric and asymmetric all-in-one SCs at a current density of 0.15 A.g^−1^: 1st, 10th, 20th, and 50th charge–discharge cycles of (**a**) SC1, (**b**) SC2, and (**c**) SC3; (**d**) 1st cycle charge–discharge curves of the fabricated SCs.

In order to test the capacitive behaviour of the above structurally characterized materials, the best suitable application is all-in-one supercapacitors. The electrochemical performance of boron carbide particles as an electrode with two different morphologies (polyhedral-equiaxed (1Y) and fiber (2Y)) were characterized in an asymmetric and two symmetric device configurations, as listed in Table 1, by employing PEIS, CV, and GCPL tests. Our previous work indicates that morphological variations strongly influence the electrochemical performance of the materials used [53]. The goal here was to compare the performance of two boron carbide-based electrodes with symmetric designs and investigate the effect of the morphological diversity between the boron carbide powders on electrochemical performance. Furthermore, with the aid of the asymmetrical design, we aimed to observe the synergetic effect of two materials on boosting the capacitive performance. Therefore, electrolyte and separator components were kept the same in all configurations. In Figure 6, Figure 7, Figure 8, Figure 9 and Figure 10, all the capacitive and electrical performance tests, as well as energy and power density dependencies, are presented.

Cyclic voltammograms (CV) were recorded at various scan rates in the range of 10–200 mV.s^−1^ to characterize the capacitive response of the fabricated SCs. Figure 6 shows the CV curves obtained from the SCs assembled with boron carbide electrodes in a two-electrode configuration from 0 to 1 V in an aqueous 6 M KOH electrolyte. The CV curves of all SCs demonstrate typical quasi-rectangular CV profiles, symmetric in charging and discharging directions, indicating EDLC behaviour with slight Faradaic contributions to the charge storage. Above 0.8 V, all CV curves showed a strong deviation from the rectangular shape of EDLC, which was expected to be observed due to exceeding the normal operating voltage of KOH electrolyte (0.8 V). The output currents for SC1 were significantly higher than those of the SC2, indicating higher specific capacitance values of SC1. The CV results show that both symmetric and asymmetric devices delivered EDLC-dominant characteristics with small Faradaic contributions.

Impedance spectroscopy is one of the most important tools to understand the kinetic processes responsible for the charge storage phenomena and the electrical resistivity of the electrodes. The Nyquist plots, the fitting parameters for the circuit elements, and the equivalent circuit obtained from the modeling of the experimental data are presented in Figure 7. The fitted Randles circuit consists of the following circuit elements: (1) resistor, which corresponds to the equivalent series resistance (R1) that includes resistances related to the electrolyte and the electrode/electrolyte interfaces in series with (2) two constant phase elements (CPE) (C2 and C3) attributed to the pseudocapacitance due to Faradaic contributions and double-layer capacitance, respectively, connected in parallel to the (3) leakage and charge transfer resistances (R2 and R3) and (4) a Warburg (W) element that represents the ion diffusion, respectively. It is worth noting that the leakage resistance is ignored since it is usually very high. As is tabulated in Figure 7d, low series resistances of 0.722 and 0.375 Ω were obtained for SC1 and SC3, respectively, whereas an increased value of 1.506 Ω was obtained for SC2. This phenomenon may be due to the different morphologies of electrode materials, that is, rapid ion transport can be achieved by the polyhedral-equiaxed morphology with smaller particle size, while the ion mobility decreases in the case of fibers. The PEIS results agree with the SEM images in which boron carbide fibers exhibit larger particle sizes, thereby leading to longer pathways for ion transport.

Figure 8 shows the energy and power densities of the symmetric and asymmetric SCs obtained from the galvanostatic charge–discharge experiments at a current density of 0.15 A.g^−1^. The asymmetric SC exhibits the highest power density of ~35 W.kg^−1^ with an energy density of 0.013 W.h.kg^−1^ (or 13 mW.h.kg^−1^) in 6 M KOH electrolyte. On the other hand, the highest energy density of 0.04 W.h.kg^−1^ (or 40 mW.h.kg^−1^) was calculated for symmetric SC1 with polyhedral-equiaxed morphology, which is approximately ten times that of the symmetric SC2 with fiber morphology and four times that of the asymmetric SC3.

A similar trend was observed in the specific capacitance results presented in Figure 9a–c. Good cycling stability was observed with average specific capacitances of 0.13, 0.01, and 0.05 F.g^−1^ at a current density of 0.10 A.g^−1^ for 50 cycles for SC1, SC2, and SC3, respectively. The details of the specific capacitance calculations can be found in previously reported studies [54,55].

Figure 10a–c display the galvanostatic charge–discharge profiles of the fabricated symmetric and asymmetric devices for the 1st, 2nd, 20th, and 50th cycles in the potential window of −1 to 1 V at a current density of 0.15 A.g^−1^. The charge–discharge profiles did not show any apparent voltage plateaus. The SC1, SC2, and SC3 delivered discharge capacities of 0.070, 0.005, and 0.025 mAh.g^−1^ and charge capacities of 0.069, 0.005, and 0.025 mAh.g^−1^, which correspond to coulombic efficiencies of 98.2, 73.6, and 99.2 %, respectively, after 50 cycles. A summary of the fabricated electrochemical performance of the symmetric and asymmetric devices is given in Table 2. These results are in great agreement with the CV and PEIS results, further confirming that the morphology has a significant effect on the electrochemical performances of boron carbide electrodes. Different morphologies of boron carbide may contain different defect structures with different concentrations, which play a direct role in enhancing the electrochemical performance of the device by providing additional pathways for ion transport [34,56]. Defects can be controlled by alternating the morphology, which can be tailored by employing different synthesis routes. As various boron carbide morphologies with different aspect ratios have different types of point defects, such as carbon or boron interstitials/vacancies, it is expected that all these point defects contributed differently to the electrochemical performance. In Figure 10, the galvanostatic discharge and charge performances of the symmetric devices composed of the same electrode material of different morphologies, i.e., polyhedral-equiaxed particles and fibers, exhibited specific discharge capacities of 0.0768 and 0.0091 mAh.g^−1^, respectively, at the 10th cycle. That is, the boron carbide electrode of polyhedral-equiaxed morphology exhibited approximately 9-fold higher specific discharge capacity than that of the boron carbide fibers at the same current density of 0.15 A.g^−1^. This is due mainly to the large difference between the particle size of the polyhedral-equiaxed particles and fibers, as depicted in Figure 5, where fibers have a much higher aspect ratio, which hinders the rapid charge transport at the electrode/electrolyte interface. The non-linearities in the E versus t curve in Figure 10d can be attributed to the Faradaic reactions contributing to the surface charge density and, hence, the surface charge reorganization.

In summary, all these electrochemical results suggest that polyhedral-equiaxed (SC1) compared with fibers (SC2) has higher electrochemical values in symmetric configurations of the supercapacitor devices. However, once the asymmetric configuration (SC3) was tested, enhancement occurred in power density and cycling stability. The obtained results indicate that the capacitive behavior of boron carbide-based electrodes is improvable by tailoring particle morphology, hence, by the aid of modifications such as doping or adding some functional groups.

## 4. Conclusions

In conclusion, an improved sol–gel approach for synthesizing boron carbide powders with various morphological features was presented without the use of catalytic elements. Particle morphology of as-synthesized powders was controlled by the optimization of thermal decomposition duration of polyol-based polymeric gel. Polyhedral-equiaxed boron carbide particles with high purity and crystallinity were synthesized by heat treatment of two-hour thermally decomposed precursors at 1500 °C for 5 h. XRD patterns of synthesized powders indicate that no carbon-based residual phases, such as amorphous carbon or graphite, remain in the powders. The morphological inspections revealed that increasing the thermal decomposition duration of polymeric gel from 2 h to 4 h resulted in a considerable morphology change, tuning from polyhedral-equiaxed to nano/micro fibers. It was found that boron carbide powders synthesized from 2 h thermally decomposed precursors have mainly polyhedral-equiaxed morphology with ~3 µm average particle size. On the other hand, increasing the thermal decomposition duration of polymeric gel from 2 h to 4 h resulted in the formation of boron carbide nano/micro fibers instead of polyhedral-equiaxed particles. The measured length and thickness of the fibers were in the range of 30 to 200 µm and sub-micron to 5 µm, respectively. The comparative electrochemical performance investigation of boron carbide polyhedral-equiaxed particles and fibers demonstrated that particle morphology has a significant effect on the electrochemical performances of the boron carbide electrodes. It was shown that the symmetric device made of electrodes with polyhedral-equiaxed morphology (SC1) exhibited rapid ion transport and yielded an excellent cycling performance with the specific capacitances of approximately 0.08 mAh.g^−1^, which is almost ten times that of SC2 composed of symmetric electrodes with fibers. Moreover, the power density and cycling stability of the device was promoted by asymmetric configuration (SC3), indicating a synergetic effect of these two morphologies.

## Figures and Tables

**Table 1:** Cell components of symmetric and asymmetric supercapacitor devices.

Device Code	Electrode Material 1 (E1)	Electrode Material 2 (E2)	Electrolyte (El)	Separator (S)	Type
SC1	1Y (polyhedral-equiaxed)	1Y (polyhedral-equiaxed)	6 M KOH	Glass fiber	Symmetric
SC2	2Y (fiber)	2Y (fiber)	6 M KOH	Glass fiber	Symmetric
SC3	1Y (polyhedral-equiaxed)	2Y (fiber)	6 M KOH	Glass fiber	Asymmetric

**Table 2:** Summary of the electrochemical performance of the fabricated symmetric and asymmetric supercapacitor devices. The cycling stability, specific capacitance and specific capacity were calculated after 50 cycles at a current density of 0.15 A.g^−1^.

Device Code	Energy Density (Wh/kg)	Power Density (W/kg)	Cycling Stability (%)	Specific Capacitance (F/g)	Specific Capacity (mAh/g)
SC1	0.045	30	84.3	0.125	0.070
SC2	0.009	27	66.2	0.009	0.005
SC3	0.013	35	90.4	0.045	0.025

## Data Availability

The data are not publicly available due to ongoing projects.
